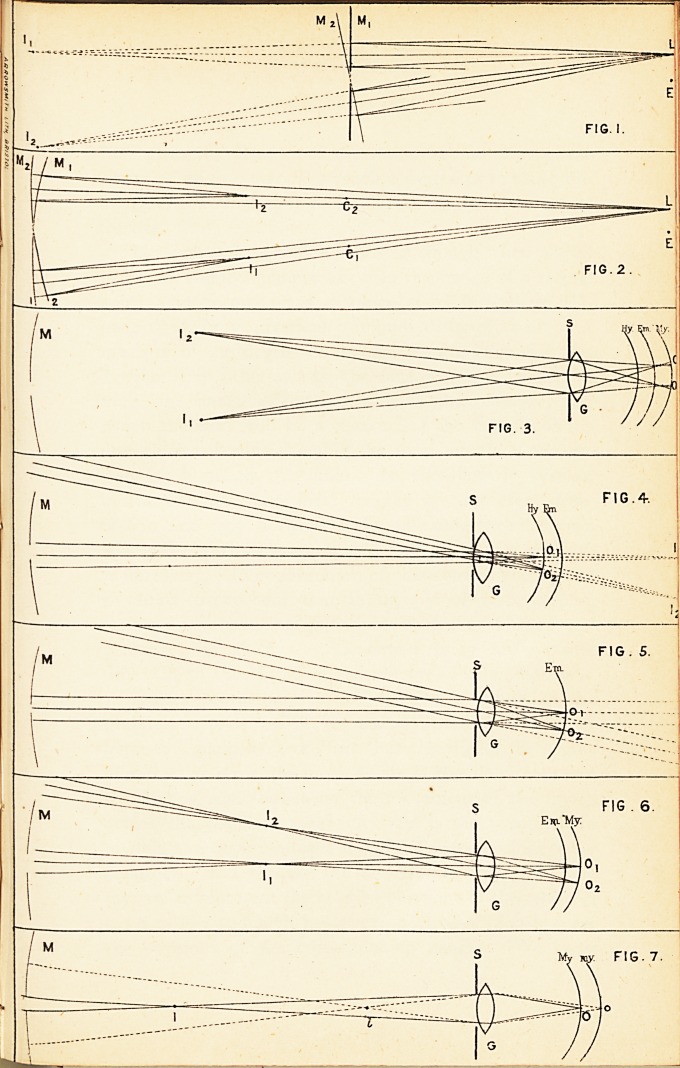# Retinoscopy

**Published:** 1883-12

**Authors:** Arthur B. Prowse

**Affiliations:** Assistant Physician to the Bristol Royal Infirmary


### RETINOSCOPY.

BY

ARTHUR B. PROWSE, M.D. Lond., F.R.C.S. Eng.,

*Assistant Physician to the Bristol Royal Infirmary.*

---

WITH the rapid progress of medical knowledge there is necessarily much more demand on the valuable time of all practitioners. A simplification, therefore, of any method of diagnosis is of great importance, and will surely be hailed with satisfaction. All should bear in mind, however, that no sacrifice of scientific accuracy must be for a moment permitted, even though a new method be one which entails less expenditure of time and trouble than the older one. Fortunately, however, we know that, in a large proportion of instances, a simpler is also a more accurate and scientific method.

Every ophthalmic specialist must often have felt the need of a readier and less troublesome mode of estimating the error of refraction in an ametropic eye than that which, until lately, has been almost universally used. More especially is this the case if the patients be children or obtuse adults, whose sensations, and their interpretation thereof, cannot be relied on as data for accurate diagnosis.

The need is felt still more when the case under examination is one of astigmatism, and not of a simply spherical error of refraction; and the number of such cases among patients of every class, and of all ages, is very much larger than is generally known. It is by no means rare for a person, the subject of astigmatism, to be assured that glasses will not benefit him; while the defect in vision is, in ignorance, attributed to some abnormal condition of the retina, or of another portion of the visual apparatus.

A method of diagnosis which would render the observer quite independent of the patient's own interpretation of his ocular sensations was a necessity, and the object of the following pages is to point out that the difficulty has been surmounted.

With this end in view I wish to bring to the notice of those members of the profession who have not had an opportunity of becoming practically acquainted with it the great value of Retinoscopy as a means of diagnosing and estimating "ocular errors of refraction."

Judging by my own experience of some hundreds of cases at the Royal Westminster Ophthalmic Hospital and elsewhere, I am quite certain that the method is very accurate if a reasonable amount of care be used, while at the same time it entails much less expenditure of time and trouble to both observer and patient.

The simple fact which forms the basis of the method is this:—If a beam of light be thrown into the eye by reflection from a mirror, the image of the illuminated, nearly circular, portion of retina with the surrounding non-illuminated part will be seen by the observer's eye (situated behind the central aperture of the mirror) to move across the pupillary area in a definite direction as the mirror is tilted about its centre; and, further, the movement of the image is in opposite directions in myopic and hypermetropic eyes.

This was known to Bowman twenty years ago, and he is mentioned in Donder's work on the *Anomalies of Refraction* as having employed it as an aid to the diagnosis of regular astigmatism at that date. Not until lately, however, has it been turned to any really practical account. In 1874 M. Cuignet, of Lille, wrote on the subject under the title of "Keratoscopy"; and a pupil of his—Dr. Mengin—introduced its practice to the notice of the profession in Paris in 1878. Dr. Parent, in 1880, published a valuable article giving the result of his investigation of the matter, and in 1881 gave some practical demonstrations of its use at Moorfields. He substituted the term "Retinoscopy" for "Keratoscopy" as being a more correct one, and recommended the use of a concave instead of a plane mirror, which until then seems to have been generally used. The method is now adopted extensively by ophthalmic specialists, and with, I believe, quite as satisfactory results as I also have obtained from it.

The reason of the concave mirror being preferable to the plane one will be at once seen by reference to Figs. 1 and 2. In these, L is the source of light; $M_1$ and $M_2$, the mirror in two successive positions; $I_1$ and $I_2$, the respective images; E is the eye; and, in Fig. 2, $C_1$ and $C_2$ are the respective centres of the curve of the mirror in its two positions. In both cases the fundus of the eye would be illuminated by rays proceeding from the image of the lamp formed by the mirror, but there is this difference in the two cases, viz., with the plane mirror the image is virtual, and is situated as far behind the mirror as the lamp is in front of it, while with the concave mirror the image is real, and situated nearer the eye than the mirror is; consequently, if both mirrors are the same distance from the eye, the illumination will be more brilliant in the latter case, and the illuminated area of the fundus will be brighter and more defined from the surrounding shade. Furthermore, it is evident that with the plane mirror the image of the lamp moves in a direction opposite to that towards which the mirror is tilted, while the reverse of this is the case with the concave reflector.

To obtain very accurate results it is usually necessary to paralyse the "accommodation" of the eye by the use of atropine (or preferably homatropine, as being more rapid and certain in its action). This is more especially necessary with children, in whom the power of "accommodation" is so great, and unless atropine has been effectually employed one is much bothered by the varying degree of myopia or hypermetropia present at different moments. Again, in those curious and interesting cases one occasionally sees of curable myopia due to spasm of the ciliary muscle, it would be almost impossible, without the use of atropine, to ascertain that the eye is generally in a hypermetropic condition when at perfect rest, *i.e.*, when no accommodative power is being exercised.

In myopic eyes, whether astigmatic or not, an approximately accurate measure of the error is usually possible without the use of atropine; but in any case of difficulty it is much better that the patient should submit to what is only a temporary inconvenience (*i.e.* the effects of atropine) rather than run the risk of wearing glasses which do not entirely correct the refractive error.

Another advantage of the preliminary use of atropine is that the dilatation of the pupil allows a better illumination of the fundus; and consequently the illuminated area and its crescentic shadowy margin are more clearly seen.

The patient should be seated in a dark room, or at all events in a dimly-lighted one, with a dark wall or screen for him to look towards. The lamp must be placed behind the vertical plane of his eyes, so that they may not be directly illuminated by it, and it may be just above, or to one side of, his head.

The other apparatus necessary is a concave mirror, with central aperture, of about 12 inches focal length, and a complete set of "trial-lenses."

The observer seats himself about four feet in front of the patient, and tells the latter to look towards a distant object just above his head; and then, by means of the mirror, reflects a beam of light into the eye under observation. If now the mirror be tilted about its centre in various directions—horizontally, vertically, and obliquely—the image of the illuminated area of the fundus with its curved shadowy border will be distinctly seen to move across the pupillary area in a direction *opposite* to that of the tilting of the mirror if the eye be hypermetropic, but in a *similar* direction if the eye be myopic.

To explain this let us refer first to Figs. 2 and 3. In the former we see that the image of the lamp in front of the mirror is the point from which the rays of light will proceed to illuminate the retina, and that this image moves in the same direction as that towards which the mirror is tilted. In Fig. 3, M, $I_1$, and $J_2$, have the same significance as in Fig. 2; G is a bi-convex lens of the same focal length as the "combined refractive media" of an emmetropic eye at rest (= 15. m.m.); S is a screen representing the iris; while the curved lines indicated by the letters Hy., Em., and My. respectively, represent diagrammatically the position of the retina in hypermetropia, emmetropia, and myopia. From the geometrical construction of the figure we at once see that the illuminated area of the retina moves in a direction the reverse of that in which the image of the lamp moves, in all eyes, whether emmetropic or not. How is it then that the observer notices a reverse movement in myopia, as compared with hypermetropia? This is easily explained by reference to Figs. 4, 5, and 6. In all these figures the lettering is the same as in Fig. 3, except that $I_1$ and $I_2$ refer, not to the images of the lamp, but to the images formed by rays of light emerging from the illuminated spots of the retina, $O_1$ and $O_2$. And it is, of course, the movement of these images that the observer sees.

In Fig. 4, illustrating hypermetropia, the object, $O_1$ or $O_2$, is between the lens and its principal focus, which is in the plane of Em.; consequently the image is virtual, and situated behind the eye, and it moves in the same direction as the illuminated area, *i.e.* in the opposite direction to the tilting of the mirror *(vide* Fig. 3).

In emmetropia (Fig. 5) the rays come from the plane of the principal focus of the lens, and after emerging are parallel, therefore a bright reflex, but no clearly defined image, will be seen by the observer; and an indistinct movement may be noticed, which, as in hypermetropia, is in the opposite direction to the tilting of the mirror.

In myopia (Fig. 6) the appearances differ according to the degree of the refractive error. In the figure the limit of distinct distant vision for small objects by the patient's eye is supposed to be less than four feet, *i.e.* the distance between observer and patient. Rays of light emerging from $O_1$ will converge to a focus at $I_1$, and similarly an image of $O_2$ will be formed at $I_2$. It is evident that in this case the image and accompanying marginal shade move in the opposite direction to the illuminated area, and therefore in the same direction as the tilting of the mirror *(vide* Fig. 3). Suppose, however, that the myopia is less in degree, so that the image of a spot on the retina would be formed more than four feet from the patient's eye. The observer will be unable to see it clearly unless he interpose a convex lens of sufficient power to bring the rays to a focus between his own "near point" of distinct vision and the patient; or unless he withdraws to a sufficiently great distance from the patient; and then, of course, the movement of the image will be in the same direction as in the higher degrees of myopia just considered. Unless one of these two proceedings be adopted the observer will see nothing but a blurred appearance, much as in emmetropia, and an ill-defined shadowy movement in a direction opposite to that of the tilting of the mirror. It will be as well here to calculate what is the highest degree of myopia which may exist and yet the image of the retinal spot be formed so far away from the patient as to be behind the observer's point of most distinct vision. If we take this as 9 inches, and he be seated 4 feet from the patient, then the furthest point from the optical centre of the patient's eye at which the observer will be able to see the image distinctly will be 4 ft. − 9 in. = $3\frac{1}{4}$ ft. (990 m.m).

Now we know that the focal length of the refractive apparatus of an eye at rest is 15 m.m., therefore from the formula for convex lenses:—

$$\frac{1}{v} - \frac{1}{u} = -\frac{1}{f} \quad \text{we obtain}$$

$$\frac{1}{v} = \frac{1}{990} - \frac{1}{15} = \frac{1-66}{990} = -\frac{65}{990}$$

$$\text{or } v = -\frac{990}{65} = -\ 15.2 \text{ m.m.}$$

ARROWSMITH LITH, BRISTOL.

FIG. 1.

FIG. 2.

FIG. 3.

FIG. 4.

FIG. 5.

FIG. 6.

FIG. 7.

This gives the distance of the retina behind the optical centre of an eye, myopic to such a degree as to cause the image of a retinal spot to be formed $3\frac{1}{4}$ feet in front of the eye.

The convex lens, which is strong enough when placed before an emmetropic eye to cause the image of a retinal spot to be formed at a distance of $3\frac{1}{4}$ ft. (990 m.m.), instead of at a practically infinite distance, will be a measure of the degree of myopia we are enquiring for. To put it in other words, the lens which is needed to make the image distinctly visible to the observer, and which therefore will be a measure of the myopia, is the one which will suffice to alter the focal length of the eye from 15 m.m. to 15.2 m.m. In the dioptric system, lenses of these two focal lengths are represented by 66.6 D and 65.7 D respectively, the difference between which is 0.9 D. Therefore a lens of about 1. D strength is necessary. In confirmation of this conclusion, we find by direct experiment that in myopia of 1. D, or less, retinoscopy shows an indistinct image, or blurred appearance, in which the movement is in an opposite direction to the tilting of the mirror, as in emmetropia.

Before proceeding to the remaining details of the practical application of retinoscopy there is one other point to explain, viz., the fact that the higher the degree of refractive error, the more feeble is the illumination of the fundus, and the slower the movement of the image across the pupillary area. As this is the case in both myopia and hypermetropia it will suffice to consider either condition. Let us take the former. In Fig. 7 My. and my. are to represent the relative positions of the retina in cases of low and high myopia respectively. I. and i. are the positions of the corresponding images. We know that the further from the optical centre of a lens the image is formed the larger will it be, and also the more quickly will any point in it move across the field of vision. Thus we at once see from the figure that the image moves more slowly in high myopia than in low, because it is nearer the optical centre of the eye. It is evident also that the much greater divergence of the rays of light in a case of high, as compared with low, myopia, causes the intensity of the illumination to diminish much more rapidly, as the distance increases, in the one case than in the other. In the figure we see that only a small beam of light passing through the lens near its centre, and through the image i., will fall on the observer's eye behind the aperture of the mirror; while if the myopia be low in degree, rays which pass through even the peripheral portion of the lens will reach the observer's eye, as well as those passing more centrally.

In a case of simple spherical error of refraction we find that the image and shadow are seen equally well in any meridian, according to the direction in which the mirror is tilted. If there be regular astigmatism this is not the case, and we are generally able to tell pretty accurately the direction of the two principal meridians by the manner in which the image moves. In simple astigmatism we find the appearances due to emmetropia in one meridian, and those due to hypermetropia, or myopia, most marked in a meridian at right angles to this. In compound astigmatism there is hypermetropia, or myopia, in both meridians, but we find that there is a distinct difference in the intensity of illumination, and in the rapidity of the movement of the image and shadow in the two directions. In mixed astigmatism there is myopia in one, and hypermetropia in the other, principal meridian. When the astigmatism is irregular in character retinoscopy is of little value; and we correspondingly find that vision is slightly, if at all, improved by glasses. Such cases are, however, comparatively rare.

When we know the *kind* of error of refraction in any eye, or in any particular meridian of the eye, we still have to estimate its *degree*. This is done by placing successively in a spectacle frame before the patient's eye lenses of different strength, commencing with a weak one, until we find that the appearances due to emmetropia have replaced those due to the kind of ametropia present. If the eye be hypermetropic we try various convex glasses, until we find one which just causes the opposite image and shadow (previously distinct) to become blurred, so that a shadowy illumination only is seen. The strength of this lens is just in excess (about 0.25 D) of the hypermetropia present. The same result will be arrived at if we use a lens which is just sufficient to replace the opposite image by one which moves in a similar direction to the tilting of the mirror, and then subtract 1. D from the strength of this lens. The remainder will be an exact expression of the degree of hypermetropia.

Suppose, now, that the eye under examination be myopic. We must use various concave lenses successively until we find one which is just strong enough to cause the similar image and shadow to become, as in the previous case, blurred and indistinct. By adding 1. D to the strength of this lens, we have a correct measure of the myopia. An alternative plan, which perhaps is preferable, especially in low myopia, is the following:—Find the lens which is just sufficient to cause the similar image and shadow to become a distinctly opposite one. The strength of this lens is just in excess (about 0.25 D) of the myopia present.

To be still more accurate it is advisable to estimate the error in both ways, and then to take the mean of the two results (if there be a difference) as expressing the degree of error.

When the myopia is below 1. D it may, without care, be mistaken for emmetropia, as the appearance of the image and shadow are alike in the two cases. But in the latter a distinctly opposite movement of image and shadow is produced by a concave lens of 0.25 D strength; whereas in the former a stronger lens than this will be necessary to produce the same result.

If an eye be astigmatic we first estimate the amount of error in one chief meridian by one of the two plans described, and then in the meridian at right angles to this.

Having thus estimated the refractive error, we must confirm our diagnosis by comparing the patient's distant vision with and without the aid of the glasses which are indicated; and while so doing, in astigmatic cases, we should be very careful to adjust accurately the direction of the axis of the cylindrical lens, especially when the astigmatism is high in degree. Finally, when atropine has been used, it is necessary to allow its effects to pass off entirely—which will take about a week—and then again to test the effect of the indicated lenses on the patient's vision for near, as well as distant, standard test-types, before proceeding to order the spectacles.

Such large numbers of people suffer inconvenience, to a greater or less degree, from the presence of unrecognised errors of refraction in one or both eyes, that I feel no apology is needed for having brought this subject once more to the notice of my professional brethren.

The relief which suitable lenses bring to such patients is so immediate and marked that I look upon this somewhat neglected field of therapeutics as worthy the most careful consideration of all who desire to do their utmost to relieve, or cure, the innumerable ills which flesh is heir to—the comparatively trivial equally with the most grave.

## Figures and Tables

**FIG. 1. FIG. 2. FIG. 3. FIG. 4. FIG. 5. FIG. 6. FIG. 7. f1:**